# Risk versus benefit: Should not audiologists assess this in the context of occupational noise-induced hearing loss in the mining industry?

**DOI:** 10.4102/sajcd.v67i2.671

**Published:** 2020-03-03

**Authors:** Katijah Khoza-Shangase, Nomfundo F. Moroe

**Affiliations:** 1 Department of Speech Pathology and Audiology, Faculty of Humanities, University of the Witwatersrand, Johannesburg, South Africa

**Keywords:** Occupational noise-induced hearing loss, Monitoring protocol, Risk, Benefit, Conservation, Vigilance

## Abstract

**Background:**

Hearing conservation programmes (HCPs) are an important aspect of occupational health efforts to prevent occupational noise-induced hearing loss (ONIHL). In low- and middle income (LAMI) countries, where the incidence of ONIHL is significant, it is important to deliberate on the risk or benefit of HCPs.

**Objectives:**

This article is an attempt at highlighting important strategic indicators as well as important variables that the occupational health and audiology community need to consider to plan efficacious HCPs within the South African mining context.

**Method:**

The current arguments are presented in the form of a viewpoint publication.

**Results:**

Occupational audiology vigilance in the form of engagement with HCPs in the mining industry has been limited within the South African research and clinical communities. When occupational audiology occurs, it is conducted by mid-level workers and paraprofessionals; and it is non-systematic, non-comprehensive and non-strategic. This is compounded by the current, unclear externally enforced accountability by several bodies, including the mining industry regulating body, with silent and/or peripheral regulation by the Health Professions Council of South Africa and the Department of Health. The lack of involvement of audiologists in the risk or benefit evaluation of HCPs during their development and monitoring process, as well as their limited involvement in the development of policies and regulations concerning ear health and safety within this population are probable reasons for this.

**Conclusions:**

Increased functioning of the regulatory body towards making the employers accountable for the elimination of ONIHL, and a more central and prominent role for audiologists in HCPs, are strongly argued for.

## Introduction

Mining plays a vital role in the growth and development of any nation in the world (Agwa-Ejon & Pradhan, 2018). In South Africa, mining and its related industries are critical for socio-economic development. In 2018, the mining sector reportedly contributed R351 billion to the South African gross domestic product (GDP) (Minerals Council South Africa, 2019). South Africa accounted for about 1.82% of the global mineral production in 2015, with a corresponding value of around US$113bn (Reichl, Schatz, & Zsak, 2017). In 2016, the mining sector in South Africa accounted for about 7.9% of the total GDP (Stats SA, 2017), which since has declined by 15% (Van Zyl, 2019). South Africa’s mineral resources have yielded, and are reported to continue to yield, significant economic wealth. Yet, decades of colonialism, apartheid, capital flight and challenges in the neoliberal post-apartheid era have resulted in high rates of occupational lung disease, hearing loss and challenges, with low rates of compensation for ex-miners (Kistnasamy et al., 2018). Given the growing advocacy and activism around occupational health issues, initiatives were launched by the South African government in 2012 towards addressing the legacy of injustice, and this included addressing minimising and/or eliminating occupational health hazards.

The African Union (AU) (2009), in its Africa Mining Vision, carefully presented a way forward for mining on the continent. The AU views this as a route towards economic development and industrialisation; however, they are very clear that this should not happen at the expense of a ‘sustainable and well-governed’ sector, which is, amongst other criteria, ‘safe’ and ‘healthy’ (African Union [AU], 2016). Coulson (2018) argued that over the last decade, the rush for economic growth on the African continent, where the pressure for job creation and poverty alleviation is palpable, can deflect attention away from the goal of a safe and healthy workplace. This author asserts that occupational health and safety (OHS) has a poor base in Africa, especially if the workplace is hazardous (Coulson, 2018). In many instances, OHS legislation is incomplete or outdated; there is a lack of enforcement by labour inspectorates, and reported data on accidents and occupational disease are low (Alli, 2008); and current authors believe this to be the case within the South African context as well.

In spite of the reported progress that has been made in combating occupational injuries, evidence suggests ongoing complex barriers and confirms the assertion by Kistnasamy et al. (2018) that there is considerable underfunding with regard to the systems required for sustained prevention and social protection (including compensation). This underfunding calls for urgent attention. These authors believe that with ‘class-action suits’ in the process of settlement, the globalised mining sector is now beginning to be held accountable. Similar trends are observed in South Africa with the recent successful landmark silicosis class-action suit, which resulted in an R5bn settlement (Niselow, 2019). This has raised the expectation of employer accountability through this critical rights-based approach, which highlights the need for a review of risks versus benefits of hearing conservation programmes (HCPs) for occupational noise-induced hearing loss (ONIHL), and the interrogation of who is well placed to conduct this risk–benefit assessment.

Occupational noise-induced hearing loss is an occupational disease (Lie, Skogstad, Johnsen, Engdahl, & Tambs, 2015), which is described as a permanent sensorineural hearing loss because of exposure to hazardous levels of noise during the performance of one’s vocation (Nelson, Nelson, Concha-Barrientos, & Fingerhut, 2005; Thorne, 2006). It is described as the second most common risk factor in the workplace, behind workplace injuries (WHO Europe, 2017). Globally, ONIHL is reported to be the number one work-related disability and the second most common form of acquired hearing loss after presbycutic hearing loss (Mostaghaci et al., 2013; Ritzel & McCrary-Quarles, 2008). In South Africa, with the high prevalence of tuberculosis (TB) and human immunodeficiency virus (HIV) or acquired immunodeficiency syndrome (AIDS) in the mining sector, ONIHL has gained prominence as an important public health priority because of these burdens of disease contributing towards the occurrence and degree of the hearing loss in this population (Khoza-Shangase, 2019). In addition, the increased attention is because ONIHL is a potentially costly public health issue, especially in a resource-constrained country like South Africa, where issues such as the minimum wage are still in the public discourse.

It has been documented that hazardous noise in the workplace results in an invisible condition, which does not readily manifest itself (Tye-Murray, 2009). In this condition, employees acquire a permanent disabling hearing loss, characterised by a hearing threshold below 40 dBs (Yadav, Yadav, Netterwala, Khan, & Desai, 2015). Copley and Frederichs (2010) and Hermanus (2007) argued that permanent disabling hearing loss is a major contributor to the global burden of disease on individuals, families, communities and countries. This burden has serious consequences for those affected.

Occupational noise-induced hearing loss has adverse consequences on the health, safety and economic outlook of the affected individuals, their families, societies and the state as well (Moroe, Khoza-Shangase, Kanji, & Nthlakana, 2018). At an individual level, ONIHL can negatively impact on the possibilities and opportunities of further employment for the affected employee (Kane-Berman, 2017). It can have a significant impact on the safety of the employee as far as work-related injuries are concerned (Amjad-Sardrudi, Dormohammadi, Golmohammadi, & Poorolajal, 2012), with prolonged exposure to hazardous noise levels in the workplace potentially leading to increased fatigue and decreased concentration, which ultimately increases human errors (Amjad-Sardrudi et al., 2012; Picard et al., 2008). Furthermore, the individual impact can spread to group effects where excessive noise exposure can potentially reduce the worker’s ability to perform or complete tasks that are significantly dependent on auditory signals or verbal communication (Thorne, 2006). This, subsequently resulting in a communication handicap, will ultimately affect team work and group productivity (Momm & Geiecker, n.d.).

At family, society and state levels, ONIHL effects are not only limited to the individual who is affected but also extend to their families, as in most cases these workers are the sole providers for or breadwinners of their families. Furthermore, this impact has an effect on the company or organisation, as these companies incur costs through compensation for OHS claims and lose productivity when employees absent from work because of this occupational injury. Lastly, because ONIHL is a disability, most workers diagnosed with a hearing loss cannot continue with their occupations. Consequently, they must rely on state resources for their upkeep as well as that of their dependants.

According to Hong, Kerr, Poling and Dhar (2013), the impact of ONIHL on one’s health and quality of life cannot be quantified in tangible measures or standards although the compensation cost for ONIHL is consistently increasing. For instance, in South Africa, ONIHL is the second most compensated occupational health condition in the mining sector (Balfour-Kaipa, 2014), second only to occupational lung disease (silicosis), when the recent lawsuit settlement is excluded. Statistics on the burden of ONIHL in developing countries are not readily available (Nelson et al., 2005); nevertheless, Chadambuka, Mususa and Muteti (2013, p. 899) argued that 80% of the individuals affected by ONIHL reside in low- and middle-income (LAMI) countries where ONIHL presents a ‘much heavier burden than in developed regions of the world’. It is for this reason that ONIHL is considered as one of the biggest threats to the country’s economy as well as public health. Kanji, Khoza-Shangase and Ntlhakana (2019) argued that current evidence about the continued existence of ONIHL as well as the reported high-compensation claims paid for ONIHL as an occupational health condition in South Africa speaks for the lack of success of HCPs in South African mines. It is for this reason that there is a need to interrogate risk–benefit assessments of HCPs within the health-resource-constrained context of the mining industry.

A key challenge with hearing conservation within the South African mining sector is that of a well-documented lack of appropriate skills as far as ear and hearing care specialists are concerned. Only a small number of South African audiologists are functioning within the occupational audiology scope of the profession, leading to unfavourable professional-to-patient ratios with obvious incongruence between demand and supply for those who do (Moroe & Khoza-Shangase, 2018). Within this sector, there is also heavy reliance on mid-level workers, such as audiometricians, by the mining industry. Another challenge is that of translating knowledge and policies into practice for various reasons, including linguistic and cultural diversity quandaries, as well as risk versus benefit assessments predicaments.

Risk–benefit assessments refer to the evaluation of safety signals (medical and/or surgical) within the health care industry, and their value has been well-documented in pharmacology (Guo et al., 2010). Such risk–benefit evaluations have not been conducted in occupational health, including in ONIHL. Because of the continued increased prevalence of ONIHL in the mining industry globally, South Africa in particular, these evaluations need increased attention from the occupational health community as part of their health and safety vigilance strategies. This article argues for increased attention to occupational audiology in Africa and the rest of LAMI countries, with clear roles for the audiologists within the team during the risk and benefit evaluation of HCPs where hazardous noise exposure has been established. Over and above the illustration of the central and leading role that audiologists should play in the process of evaluating risk versus benefit in HCPs, which this article aims to do, the centrally located and more visible role of the Minerals Council of South Africa (previously Chamber of Mines) within the multidisciplinary and multi-stakeholder team in ensuring adherence to regulations has been discussed.

The Mine Health and Safety Council (MHSC) has ‘every mine worker returning from work unharmed everyday: Striving for zero harm’ as one of its key goals (MHSC, 2016). In spite of all documented efforts by the MHSC and the Minerals Council South Africa to achieve this goal as far as ear and hearing health is concerned, this goal has not been achieved as yet (Booyens, 2013). Evidence suggests that over 70% of miners in South Africa are exposed to excessive noise levels, well above the legislated occupational exposure limit of 85 dBs, in spite of the presence of HCPs in this sector (Edwards, Dekker, Franz, van Dyk, & Banyini, 2011; Strauss, Swanepoel, Becker, Eloff, & Hall, 2012). Efficacy of HCPs has been found to be limited to non-existent, with published South African studies revealing that HCPs have not been successful in the mining industry. After the formal implementation of HCPs in 1996, subsequent to the promulgation of the *Mine Health and Safety Act of 1996*, numerous studies that have been conducted to evaluate the effectiveness and efficacy of management of ONIHL within the South African mining industry have yielded findings that indicate little, if any, success with HCPs (Edwards, Milanzi, Khoza, Letsoalo, & Zungu, 2015; Edwards et al., 2011; Edwards & Kritzinger, 2012; Kanji et al., 2019; Moroe, 2018; Moroe & Khoza-Shangase, 2018; Ntlhakana, Kanji, & Khoza-Shangase, 2015; Strauss et al., 2012).

## Evaluation of risk versus benefit

The obvious goal in HCPs is to recognise the harmful effects of excessive noise exposure by qualified professionals, utilising objective measures, before any serious injury is experienced by employees (Byrne, 2005). In addition, this goal extends to the elimination and/or minimisation of harmful noise (Byrne, 2005) through a hierarchy of controls that include elimination of the hazard, engineering controls, substitution of equipment and administrative controls before the provision of hearing protection devices. Unfortunately, HCPs and ONIHL identification, and monitoring protocols have not been standardised or strictly adhered to nationally as well as internationally; therefore, with limited to no evidence of industry best-practice sharing, final analyses of their efficacy are not always entirely dependable and/or generalisable. More importantly, the role of the audiologist, whose scope of practice HCPs falls under, is not prominent within the South African occupational health industry. It is for this reason that the audiology community needs to directly engage in the development of policies and regulations around HCPs, the development process of the HCPs as well as in the implementation and monitoring of these programmes (Moroe, 2018).

The current authors assert that comparative evaluation of benefits (positive effects – efficacy) and risks (potential harm) of HCPs within the South African mining industry is of paramount importance in occupational health. One would argue that the responsibility for this evaluation lies with the mining companies (employers) whose benefit or risk monitoring of their HCPs should be ongoing, taking careful cognisance of contextual factors such as burden of disease in their workforce. The goal would be to ensure that once a risk assessment for noise has been carried out, minimisation, if elimination is not possible, of safety hazards and maximisation of HCPs benefits ensues.

The Health and Safety Authority (n.d.) highlights that regulation 124 states that determination and assessment of risks must be carried out by the employer, and that during this task, the employer must ensure that the risk assessment meets the requirements of the regulations. This assessment must be conducted:

- When employees are liable to be exposed to noise at work, which is above lower levels of exposure.
- When employees are exposed to levels of noise whose safety or health is particularly at risk.
- When any effects on employees’ safety and health result from any interactions between noise and work-related ototoxic substances, and between noise and vibrations.
- When there is an indirect effect on employees’ safety or health from interaction between noise and warning signals, or other sounds that need to be observed to reduce risks of accidents.
- When information on noise emission is provided by the manufacture of work equipment, in accordance with section 16 of the *Safety Health and Welfare at Work Act* 2005.
- If there is an extension of noise exposure beyond the normal working hours. This comes under the employer’s responsibility, or the action value, and the employer must consult with his or her employees or their representative and make an appropriate assessment to reduce the risks involved.
- When alternative equipment is made available to reduce noise emission.

Close analysis of the afore-listed occasions as to when risk assessment should take place, and by whom, reveals budgetary needs, which the current authors argue would sharply raise possible conflict of interest if there is no independent external risk assessor and strict accountability checking by the regulator. These costs extend to remediation requirements, for example, the availability of alternative equipment, which might be required to reduce the noise emission levels, availability and use of ear protection, as well as costs for review of risk assessment where the results of health surveillance show it to be necessary – as per Regulation 131.

The remediation steps that the employer can take to prevent or control the risks associated with ONIHL, as with any other hazard, fall under a hierarchy of control options that include elimination, substitution, personal protection equipment (PPE) and so on. Specifically, noise elimination and control can be seen as engineering (e.g. control of vibration by damping or tightening parts in the noise source), administrative (e.g. by good procurement or ‘buying quiet’ or by rescheduling work to decrease exposure time of the employees involved) and personal protection (as a last resort by the use of suitably selected personal ear protection). The costs involved in removing the source of noise from the workplace, controlling the noise at source, implementing collective control measures (e.g. by engineering controls, such as enclosing the noise source; workplace design, such as isolating the noise source; or having suitable acoustics within the work area to reduce the transmission of noise) and supplying individual control measures (personal protective equipment) if the measures above are not adequate as an interim measure are the responsibility of the employer – and this has implications for the company’s bottom line. This is where the current authors identify the consequent introduction of a conflict of interest should the employer be expected to self-regulate; as is currently the expectation. The fact that companies use PPE (in the form of personal hearing protection devices) as their main hearing conservation measure, while failing to meet the basic rule of making the workplace as quiet as possible first, is an example of cost-cutting measures fuelled by conflict of interest of the employers. The current authors wish to raise a flag of caution about this conflict and argue that this situation is possibly at the core of the reasons why elimination of ONIHL milestones is not being met within the South African context. It therefore becomes critical that such benefit or risk evaluations of HCPs are comprehensive, inclusive and ongoing with representation of all stakeholders – but with management and leadership from the regulating body – externally to and independent of the employer.

Standard HCPs consist of six components designed to reduce the level of noise and to identify other ways to protect workers from ONIHL. These are: noise measurement; education and training for workers; engineered noise control; hearing protection devices; hazard awareness, including warning signs; and hearing tests for workers (Amedofu, 2007; Hong et al., 2013). Over and above the conflict of interest issues raised earlier, the absence of audiologists as key members of the team in South African HCPs raises important implications for ONIHL and HCPs where ongoing benefit or risk evaluations are conducted.

Khoza-Shangase (2017), in conducting the same exercise linked to pharmacovigilance, argued that benefit or risk evaluation should, at a minimum, be conducted by researchers (including those in the engineering field); physicians or clinicians or audiologists acting on behalf of their patients (employees); the patients themselves (employees or their unions’ representatives); and the regulatory authority, which decides on whether an HCP should be approved or not, whether it should be reviewed or withdrawn or not, while making recommendations about the alternative ONIHL-preventative measures for intermediate action. As in pharmacovigilance within the South African context, numerous challenges can be identified with each of these groupings responsible for this benefit or risk evaluation, particularly within the developing country context with unlimited employer–employee challenges and enforcement of regulation barriers.

## Factors influencing benefit or risk evaluation in hearing conservation programmes

Within the South African context, benefit or risk evaluation can be significantly impacted by factors such as nature of the problem and its impact, indication for HCPs and population under the programme, economic factors (such as costs involved, including compensation claims), stakeholders with vested interests in the HCP, as well as time, data and resources constraints. Firstly, the nature of the problem that determines the time course of action, which usually defines the socio-medical seriousness of the suspected adverse noise exposure reaction and its potential threat to life, can be limiting when it comes to the inclusion of important quality of life side effects such as ONIHL. This is particularly so because ONIHL is a silent and invisible disability that, in this case, afflicts mostly the already vulnerable members of the society (poor uneducated older black men, who are sometimes immigrants from neighbouring countries). Consequently, quality of life in the benefit or risk evaluation becomes less prioritised and requires much deliberation in the planning, implementation and monitoring of HCPs.

Secondly, indication for HCPs and the population under the programme also influences the benefit or risk evaluation. Audiologists and regulators working in mines’ HCPs need to be aware of the risks in the workplace (established through the determination and assessment of risks), factors influencing risks (such as age, burden of disease, exposure to concomitant risks to ONIHL and so on) and be aware of all circumstances where there are no reasonable elimination alternatives, and plan their HCPs appropriately. For example, employees on treatment for TB at risk for ototoxic hearing loss are at an even greater risk for more significant ONIHL as the two ear toxins (noise and ototoxic medications) act synergistically; therefore, an HCP for these employees needs to take this into consideration. Such HCPs should acknowledge that HCPs are complex interventions, as suggested by Moroe (2018), and adopt more individualised strategies where required. When elimination is not possible, carefully planned and executed education and training of employees around hearing conservation with appropriate use of PPEs should be provided, with stringent monitoring and early intervention strategies in place.

Thirdly, economic factors (such as costs involved, including compensation claims), particularly in LAMI countries like South Africa, play a major role in benefit or risk evaluations (Khoza-Shangase, 2017). Employers, in their concern about their bottom line quest for high profits, might accept health and safety conditions with less favourable benefit–risk balance because of the drive for profits as well as affordability of the HCP, when compared to the alternative; for example, ‘buying quiet’ might be more expensive than paying compensation for ONIHL. This is where the lobbyist role of audiologists should become heightened and the regulatory role of the Minerals Council South Africa should be enforced, where mining companies are directly engaged with to ensure ethical accountability around employees’ health and safety where both profits (productivity) and employees’ quality of life are taken careful cognisance of. Economic factors in benefit or risk evaluation are concerned with trade-offs and weighings where economic efficiency in terms of the difference between benefits and costs is measured (Khoza-Shangase, 2017). If quality of life’s ‘side-effects monitoring’ such as annual audiometric monitoring assessments did not form part of the HCP development process, then economic factors might be miscalculated. For ONIHL, permanent long-term hearing loss brings with it significant economic costs to both the patient and the state if not prevented and/or eliminated. Firstly, loss of employment because of a hearing loss has implications for the economy of the country, and secondly, rehabilitation costs such as fitting of hearing aids and aural habilitation cannot be underestimated. This is over and above the compensation claims that the companies have to pay for ONIHL.

Fourthly, stakeholders with vested interests in the HCP, such as employees, occupational health physicians, mining companies, academics, ethics and health and safety committees, regulatory authorities, other public health bodies, compensation funds, labour and trade unions, may all have very different perspectives on benefit or risk evaluation of HCPs. It is therefore important to be aware of who the stakeholders were during the determination of the benefit or risk of an HCP. As practicing clinicians and audiologists, the employees’ perspective seems the logical site for evidence base for objective collection of audiological data for ONIHL. In the South African context, sensitivity towards the influences of language and cultural diversity as well as the demographic and medical profile of employees to clinical management involved in HCPs is important. Two employees exposed to the same benefits and risks may view the risk differently, may be affected by the risk differently, may accept the risk differently and may also make different decisions around the risk. Influences of language and cultural diversity may compound this level of risk evaluation. Nonetheless, it is important that such a risk is clearly communicated to the employees not only for informed consent but also because proper pre-exposure counselling and education about side effects of excessive noise has been shown to improve adherence to safety measures, such as use of PPEs (Balkhyour, Ahmad, & Rehan, 2019).

Lastly, time, data and resources constraints significantly influence benefit or risk evaluation (Council for International Organizations of Medical Sciences [CIOMS], 1998). This is particularly important when the potential major risk is urgent. As ONIHL may not be considered a major occupational injury, the time urgency may not be taken seriously. However, it is important that sufficient data on comparator HCPs or other hearing conservation strategies be obtained as early and as reasonably quickly as possible. That means accurate and sensitive standard hearing as well as noise measurement and monitoring protocols need to be implemented in all sites where noise levels above regulated levels are present. Systematic, comprehensive and standardised HCPs would facilitate collation of large databases that would allow for an easy review of ONIHL and the context and conditions surrounding it. The current status of HCPs in South Africa, which consists of non-standard and semi-regulated monitoring programmes, mostly conducted and managed by people whose jobs fall outside the scope of HCPs, would negatively influence benefit or risk evaluation of any programme component. Besides standardising the monitoring protocols, the audiology community as well as the regulating body would also need to address the issue of manpower and equipment constraints that are major contributors to the limited and less successful HCPs in the South African context. Within the South African context, when there are limited audiologists to population size, with an even lesser engagement of audiologists in occupational health settings, careful consideration about utilising trained non-audiologist screeners to implement HCPs under strict audiologist management, as per regulations, need to be made. Furthermore, serious deliberations around the use of tele-audiology to increase access, quality and control need to be undertaken.

## What the regulator and audiologists need to consider

For benefit or risk evaluation in HCPs, the regulator and the audiology community need to carefully engage in scoping the context, benefit evaluation, risk evaluation, benefit–risk evaluation, options analysis, as well as deciding of the available options, which are appropriate for the context.

Firstly, as far as scoping the context is concerned, each context needs to establish the risk profile of the mine’s context as well as the specifications or descriptions of the HCP implemented within their context. This needs to be continuously updated as new risks (e.g. introduction of additional toxins such as ototoxic medications) and/or new noise-generating equipment or Hearing Protection Devices (HPDs) are introduced in the mine. Establishing indications for use and review of HCPs based on the factors, such as levels of noise, burden of disease as well as the various shifts within the mining industry, is crucial. Once indications have been established, identification of one or more alternative preventive measures (including ‘buying quiet’) forms part of the important considerations. Lastly, scoping the context also includes establishing and providing descriptions of the suspected or established ONIHL diagnosis. Establishing the time, the degree and the progression of tinnitus and hearing loss is important.

Secondly, benefit evaluation is another important consideration to be made during benefit–risk evaluation. The current authors strongly believe that this should be led by the regulating body. This evaluation involves studies of the incidence or prevalence and the natural history of the target disease (Khoza-Shangase, 2017) – in this case ONIHL. It also entails establishing the purpose of HCPs, and how this influences the occurrence of ONIHL in this population. Furthermore, benefit evaluation involves comparison of information on efficacy and general toleration data toalternative preventive measures. These alternatives include the use of other strategies, such as pharmacotherapeutic intervention strategies namely, the use of oto-protective agents as well as the option of elimination of noise.

Thirdly, an area where audiologists should be significantly involved is risk evaluation. Risk evaluation is another consideration where the weight of evidence for the suspected risk (ONIHL) in terms of incidence and/or prevalence is established. It is also the process where ‘risk profiles’ with their most common reactions to the target HCP as well as similar profiles for alternative HCPs are drawn up, and comparisons are made. This requires proper record-keeping as well as the use of standardised protocols that will allow for proper and accurate comparisons. Risk evaluation for ONIHL would include detailed presentations and analyses of evidence such as audiograms, Distortion Product (DP)-grams and noise-level measurements, where extraneous variables such as concomitant treatments (e.g. in HIV or TB treatments), concomitant presbycutic hearing loss and so on are either eliminated or carefully controlled for. Considerations of preventability, predictability and reversibility of ONIHL also form part of risk evaluation. Strategies such as the use of PPEs, oto-protectors, less ototoxic medications, where such medications are prescribed for medical conditions such as TB in employees, redeployment to less noisy environments when the risk is higher, shift manipulations and so on become key during this process.

Fourthly, all the above-listed considerations allow for benefit–risk evaluation where the purpose and effectiveness of HCPs as well as benefits related to the seriousness of ONIHL are summarised. Moreover, dominant risks and their severity, duration and incidence are also summarised. Lastly, taking into account alternative preventive measures, the benefit–risk relationship is also summarised. This process can be properly performed only if evidence with regard to appropriate medical, audiological, engineering and administrative controls has been collated, analysed and presented during the establishment of the benefit–risk relationship.

Lastly, once the risk–benefit relationship has been established, it is important for audiologists to engage in options analysis where a list of all appropriate options for action is determined. This is where the pros and cons and the likely consequences (impact analysis) of each option under consideration are described, and the manner in which the consequences of the recommended action should be monitored or assessed is suggested (CIOMS, 1998). It is anticipated that this is the process that would be the most challenging for audiologists in this industry where available options are significantly influenced by financial implications for the companies involved, as well as demand-capacity challenges. These available options include maintenance of the *status quo* when there is no evidence for ONIHL concern; ‘watching and waiting’ which involves monitoring subsequent experience of ONIHL if insufficient evidence exists and additional data are gathered; intensive additional data gathering or new research that can be clinical or non-clinical involving standardised protocols for data collection in clinical and non-clinical settings; modifications to the HCPs or their use or amendments to the information on HCP components; suspension of HCP licence if found not to be efficacious; withdrawal of the HCP from the specific mine, which can be voluntary or mandatory; as well as communication of new or reinforced information to the regulating body, the mines, the occupational health profession or the employees about the HCP.

## Decision-making: Principles to consider when deciding on the options

Decision-making during benefit–risk evaluation needs to be conducted by a multidisciplinary and/or multi-stakeholder team guided by principles including objectivity, equity and accountability (CIOMS, 1998; Khoza-Shangase, 2017). Objectivity, firstly, refers to objectivity of the evidence base for all relevant sources by a variety of methods (observational, epidemiological and experimental) and evidence gathered by people who are not conflicted with regard to conflict of interest. Secondly, it refers to expertise, which is led by the regulator and includes the employers as well as the employees. The principle of objectivity ensures avoidance of bias and conflict of interest. It calls for scientific decision-making such as use of algorithms and matrices, and it ensures that decision-making is based on explicit predetermined criteria (Khoza-Shangase, 2017; Tsintis & La Mache, 2004). The audiology community would need to establish and standardise monitoring protocols that can facilitate objectivity in decision-making.

As far as the principle of equity is concerned, all evidence-based HCPs should be treated fairly. This principle dictates that decision-making must be transparent, sensitive and specific; follow due process; be an open process; and involve consultation with relevant stakeholders, utilising an appropriate comparator (Khoza-Shangase, 2017). The principle of accountability highlights that the expected outcomes must be specified or estimated; that criteria should be established for determining and assessing the effectiveness of the actions chosen; and that if action was not total elimination of noise, data must continue to be collected for ongoing monitoring of safety.

## Immediate plan: What audiologists should do

Audiologists need to make sure that when excessive occupational noise exposure is present, pre-exposure education to ensure awareness and therefore use of PPEs is in place. This is after the hierarchy of controls has been observed. In spite of the fact that there are currently limited alternative options where elimination is not possible or has not been implemented for an employee who is desperate for employment, it still remains an ethical obligation to inform the employees of potential harm in the form of ONIHL. This has the benefit of facilitating prevention and/or early identification of ONIHL because the employee will be aware of, and adhere to, HCP strategies, and will also be encouraged to attend monitoring sessions. Furthermore, pre-exposure education about possible impacts of noise has a positive impact on programme adherence, as employees will be less likely to default on preventive measures because of unknown, unexplained or unanticipated signs and symptoms.

Regardless of the fact as to whether typically permanent threshold shift has occurred, the cochlea cannot recover and so early identification of ONIHL is crucial. Along with rimary prevention, early detection of hearing loss is important for providing management options, such as changing or modifying of hearing protection devices to potentially more effective, custom-made HPDs; manipulating shifts and exposure times for the employees involved; planning audiological management and counselling; complete removal from noise if synergistic effects of ototoxicity and noise are at play; amplification in cases of severe hearing loss; compensation, among others

As far as alternative medical intervention is concerned, audiologists, physicians and pharmaceutical companies should intensify their efforts towards development and building the evidence base for oto-protective agents, which can serve as a preventative measure where excessive noise exposure cannot be avoided. Oto-protective agents in the form of compounds, such as vitamins A, C and E (ACE) magnesium, D-methionine (sulphur-containing compound) and L-N-acetylcysteine, should be investigated (Le Prell et al., 2014). Furthermore, restorative care that involves regeneration of hair cells damaged by toxins through the use of neurotrophins also needs careful consideration (Wissink, Moes, Beisel, & Fritzsch, 2006). These strategies are especially important in LAMI country contexts for strategic long-term financial savings that will be made by eliminating potential litigation costs, amplification device costs, rehabilitation costs as well as social-grants-linked costs because of the economic impact associated with unemployment of the affected individual (Khoza-Shangase, 2017).

## Future directions

The regulating body needs to carefully deliberate on its role in regulating adherence to regulations around occupational health in the South African mining industry. The current player or referee role that the mining industry holds when it comes to health and safety of its employees needs to be reviewed – with external independent accountability in place.

For large sets of data that can be used to establish evidence that is enough for benefit or risk evaluation in HCPs, audiologists need to engage in occupational audiology studies where ONIHL vigilance is prioritised in order to meet the elimination targets. This vigilance should take careful cognisance of the influence of the burden of disease, such as ototoxicity, because of TB in the mining population.

While the above studies will involve currently implemented HCPs, parallel studies on alternative HCPs, including ‘buying quiet’ and using oto-protective agents, should also be run in order to allow for the development of lists of options for ethical benefit or risk assessments. This is important because establishing benefit or risk without any alternative options can be argued to be an unethical practice. Also continuing to expose employees to noise levels with well-established ear and hearing effects because there are no alternatives is not best practice, and it impinges on the employees’ human rights.

Audiologists’ active and strategic involvement in HCPs, through establishing hearing, monitoring and educational programmes, would facilitate the establishment of evidence in these contexts to demonstrate the important role that the audiologists have in both the assessment and treatment of employees at risk for ONIHL. Their active involvement in task teams putting together HCP guidelines, using evidence base to continuously inform policy, would ensure that this often neglected disability is also considered in the benefit or risk evaluation processes. Audiologists’ role in lobbying and advocating for their expanded role in health and safety programmes, HCPs approval, and HCPs implementation and monitoring is paramount for a useful engagement in benefit–risk assessment for all HCPs. Involvement in advisory panels on benefit, risk and cost management of HCP components where the risk of ONIHL is a reality, as well as providing recommendations on communication and awareness programmes for the employees on ONIHL, should form part of the workload for audiologists involved in HCPs. All this can be performed through employing various service delivery models, including the use of tele-audiology and task shifting. Risk versus benefit assessments in this context should be guided by the amount of risk the employees are exposed to as well as the contributing factors. Therefore, exposure quantification as well as noise level measurements are key in determining the timeline or frequency as to when the risk or benefit evaluations should be performed (biannually, annually, etc.). At a bare minimum, risk or benefits should be performed annually within a dynamic risk assessment approach, with audiologists playing a key role in leading and managing the process.
